# Prospective evaluation of NGS-based sequencing in epilepsy patients: results of seven NASGE-associated diagnostic laboratories

**DOI:** 10.3389/fneur.2023.1276238

**Published:** 2023-12-06

**Authors:** Maximilian G. W. Witzel, Christian Gebhard, Sören Wenzel, Saskia Kleier, Birgit Eichhorn, Peter Lorenz, Laura von der Heyden, Marius Kuhn, Manuel Luedeke, Miriam Döcker, Jerome Jüngling, Björn Schulte, Konstanze Hörtnagel, Ralf Glaubitz, Sarah Knippenberger, Anna Teubert, Angela Abicht, Teresa M. Neuhann

**Affiliations:** ^1^MGZ Medizinisch Genetisches Zentrum, München, Germany; ^2^Gemeinschaftspraxis für Humangenetik and Genetische Labore Hamburg, Hamburg, Germany; ^3^MVZ Mitteldeutscher Praxisverbund Humangenetik GmbH, Dresden, Germany; ^4^MVZ Genetikum GmbH, Neu-Ulm, Germany; ^5^Zentrum für Humangenetik, Tübingen, Germany; ^6^Zentrum für Humangenetik und Laboratoriumsdiagnostik (MVZ), Martinsried, Germany; ^7^Amedes Genetics, Hanover, Germany

**Keywords:** epilepsy, pediatric, neurology, syndromic, genetics, next-generation sequencing

## Abstract

**Background:**

Epilepsy is one of the most common and disabling neurological disorders. It is highly prevalent in children with neurodevelopmental delay and syndromic diseases. However, epilepsy can also be the only disease-determining symptom. The exact molecular diagnosis is essential to determine prognosis, comorbidity, and probability of recurrence, and to inform therapeutic decisions.

**Methods and materials:**

Here, we describe a prospective cohort study of patients with epilepsy evaluated in seven diagnostic outpatient centers in Germany. Over a period of 2 months, 07/2022 through 08/2022, 304 patients (317 returned result) with seizure-related human phenotype ontology (HPO) were analyzed. Evaluated data included molecular results, phenotype (syndromic and non-syndromic), and sequencing methods.

**Results:**

Single exome sequencing (SE) was applied in half of all patients, followed by panel (P) testing (36%) and trio exome sequencing (TE) (14%). Overall, a pathogenic variant (PV) (ACMG cl. 4/5) was identified in 22%; furthermore, a significant number of patients (12%) carried a reported clinically meaningful variant of unknown significance (VUS). The average diagnostic yield in patients ≤ 12 y was higher compared to patients >12 y cf. **Figure 2B** vs. **Figure 3B**. This effect was more pronounced in cases, where TE was applied in patients ≤ 12 vs. >12 y [PV (PV + VUS): patients ≤ 12 y: 35% (47%), patients > 12 y: 20% (40%)]. The highest diagnostic yield was achieved by TE in syndromic patients within the age group ≤ 12 y (ACMG classes 4/5 40%). In addition, TE vs. SE had a tendency to result in less VUS in patients ≤ 12 y [SE: 19% (22/117) VUS; TE: 17% (6/36) VUS] but not in patients >12 y [SE: 19% (8/42) VUS; TE: 20% (2/10) VUS]. Finally, diagnostic findings in patients with syndromic vs. non-syndromic symptoms revealed a significant overlap of frequent causes of monogenic epilepsies, including *SCN1A, CACNA1A*, and *SETD1B*, confirming the heterogeneity of the associated conditions.

**Conclusion:**

In patients with seizures—regardless of the detailed phenotype—a monogenic cause can be frequently identified, often implying a possible change in therapeutic action (36.7% (37/109) of PV/VUS variants); this justifies early and broad application of genetic testing. Our data suggest that the diagnostic yield is highest in exome or trio-exome-based testing, resulting in a molecular diagnosis within 3 weeks, with profound implications for therapeutic strategies and for counseling families and patients regarding prognosis and recurrence risk.

## 1 Introduction

Epilepsy is one of the most common and disabling neurological disorders. Overlapping syndromic and non-syndromic phenotypes often preclude a purely clinical diagnosis and prevent targeted genetic testing, i.e., gene by gene (1). Over the past 2 decades, numerous genes have been identified that are associated with epilepsy; with modern sequencing technologies widely available in research and diagnostics, the amount of confirmed monogenic epilepsy disorders is increasing rapidly. Studies suggest that in more than 30% of patients, molecular diagnosis has possible precision medicine implications (2). In addition, the knowledge of the molecular basis has an impact on determining the prognosis, comorbidity, and probability of recurrence. Therefore, molecular genetic testing has been implemented in many clinical workup processes for epilepsies.

Monogenic epilepsies can be characterized according to the gene function affected: Impaired function of ion channels (e.g., CACNA1A), receptors (e.g., GABRB3), transporters (e.g., SLC2A1), synapse-related (e.g., PRRT2), and other pathways (e.g., CDKL5, PCDH19) are associated with epilepsy. Genes related to mechanisms of cell growth, division, and proliferation (e.g., DEPDC5, MTOR, TSC1), as well as genes related to cell metabolism (e.g., OTC), protein biosynthesis (e.g., CLN6), and mitochondrial function (e.g., POLG) can be distinguished.

Monogenetic epilepsy phenotypes can also be classified by phenotype, e.g., generalized and focal epilepsy syndromes.

Focal epilepsies include those with anatomical anomalies, e.g., COL4A1-related porencephalic cysts or GNAQ mosaic mutations causing Sturge–Weber syndrome.

A particularly high yield is reported for early epileptic infantile encephalopathies (EEIE) and children with developmental delay and encephalopathy, which clinically overlap to a large extent. In this group of patients mostly autosomal dominant *de novo* variants are identified, whereas autosomal recessive traits are less common (3, 4). Developmental and epileptic encephalopathies (DEE/EEs) are characterized by early onset of seizures, therapy resistance, neurodevelopmental delay, for example, as seen in channelopathies (e.g., GRIN2A-, SCN2A-, SCN1A-, and SCN8A-related epilepsies) or in metabolic conditions (e.g., SLC2A1).

Out of these epilepsy phenotypes, some more common forms emerge. Pathogenic PRRT2 variants cause self-limited (familial) infantile epilepsy (SeLIE) among other pleiotropic syndromes. PRRT2-related epilepsy is the most common monogenic epilepsy with an incidence of 1 per 9,970 live births.

In addition, Dravet syndrome due to pathogenic variants in SCN1A, KCNQ2-related epilepsy early-onset DEE due to loss-of-function mutations in KCNQ2, GLUT1 deficiency syndrome, due to pathogenic variants in SLC2A1, CDKL5 pathogenic variants causing a severe early onset DEE, PCDH19 girls-clustering epilepsy (PCDH19-GCE), SLC6A1-associated DEE and TSC1- and TSC2-associated epilepsy as in tuberous sclerosis are well-known causes of epilepsy syndromes in the neuropediatric clinic [summarized and adapted from Guerrini et al. (5).

The purpose of this prospective study was to evaluate the diagnostic yield of panel (P), single-exome (SE) and trio-exome sequencing (TE) in an unselected cohort of patients with seizures to draw conclusions for clinical testing strategies. This included patients of all ages, regardless of epileptic semiology and with or without additional syndromic findings.

## 2 Methods

All patients received diagnostic testing for epilepsy as ordered by their attending physicians (e.g., neurologist, pediatrician, and geneticist) at one of the above-mentioned, seven diagnostic centers in Germany.

After genetic counseling, informed consent was signed and obtained in all cases. Patients agreed to the exchange of pseudonymized data in a scientific context. Diagnostic labs shared data in common genetic databases, e.g., LOVD and ClinVar (e.g., MGZ).

All samples that were evaluated (e.g., for which a report was provided) over a period of 2 months—from July 2022 to August 2022—from patients with a seizure-related human phenotype ontology (HPO) term (e.g., Seizure HP:0001250 and Status epilepticus HP:0002133) and which received a next-generation sequencing-based genetic testing were included. Genetic testing was either performed as panel-testing (P), single exome (SE) or trio exome (TE).

Actionability or changes in clinical management for patients with diagnostic findings (PV + VUS) were inferred from a previously published data set of 25 genes (6) Supplementary Table 1. We compared these 25 genes plus the *POLG*-gene with findings in our cohort, by percentage and counted numbers of cases with actionable findings (a total of 26 actionable genes). The reason to include *POLG* was that it is a frequent request to genetic laboratories to rule-out POLG pathogenic variants in order to avoid valproate (if necessary) and to provide an indication for specific treatments (e.g., EPI-743, vatiquinone). Of note, this constitutes the lower threshold of actionability since diagnostic results in other gene might still influence the medical decisions albeit not included in the above-mentioned data set by McKnight and Bristow, or non-therapeutic decisions are achieved (e.g., avoidance of invasive diagnostics, counseling). Individual follow-up information is not available for our cohort.

Statistic evaluation and data visualization was performed using msOffice Excel and R-Software environment (RRID: SCR_001905) (7) with packages ggplot2 3.4.0 (8) (RRID:SCR_01460), ggdist (3.3.0) (9) gghalves (0.1.4.) (10), treemapify (2.5.6) (11), and geomtextpath (0.1.1) (Cameron & Brand, o. J.).

Venn-Diagrams were created using https://bioinfogp.cnb.csic.es/tools/venny/ Venny 2.1 (RRID: SCR_016561).

For the evaluation, patients were divided into age groups (≤12 y, >12 y) and phenotype. Patients' phenotypes were classified as syndromic or non-syndromic based on clinical information. Symptoms and clinicopathological comorbidities other than epilepsy and/or epileptic encephalopathic disorders were classified as “syndromic,” whereas only seizure-related findings were classified as “non-syndromic.”

The respective patient groups were evaluated for diagnostic yield, the molecular result, sequencing method, and turnaround time (TAT)—calculated as time between sample entry and date of report. In each group, the proportions of individuals with a diagnostic finding [variant of unknown significance (VUS), pathogenic variant (PV)] or non-diagnostic finding were calculated.

## 3 Results

**Figure 1:**
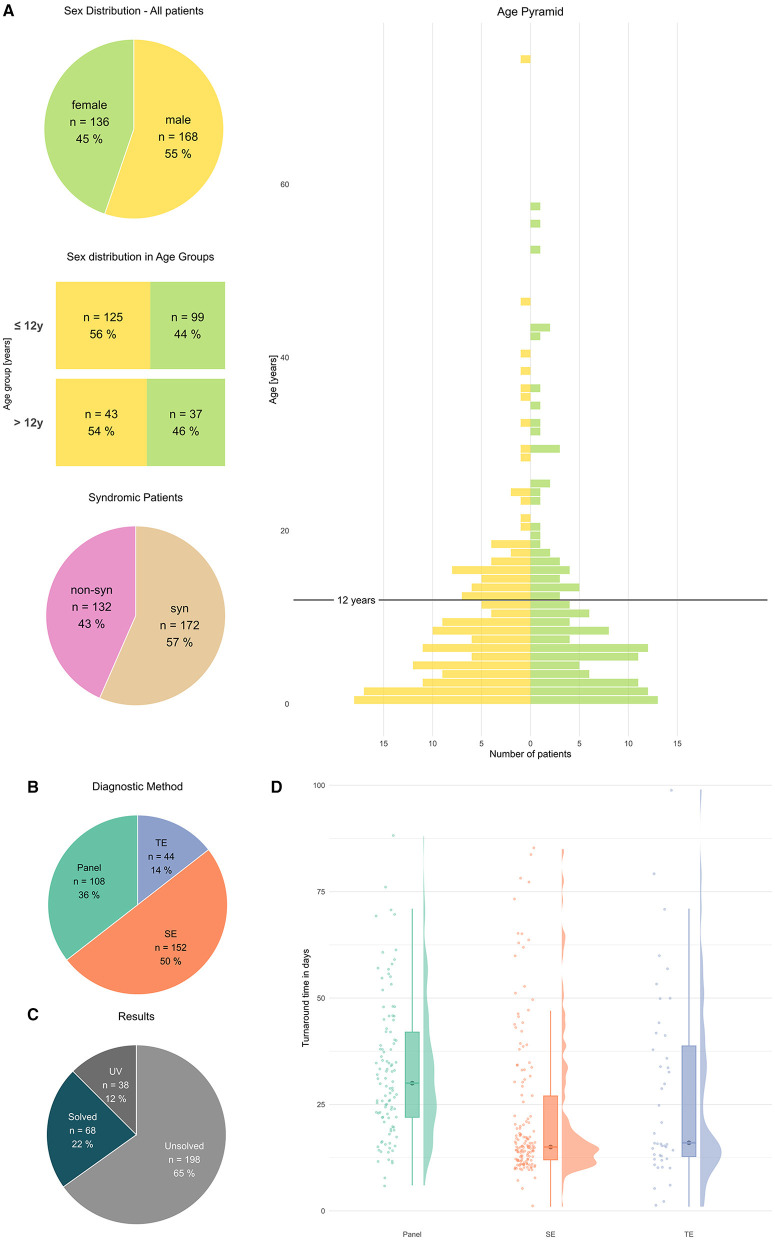
General characteristics of patients and methods. **(A)** Patients characterized by sex, sex distribution in age groups, and syndromic feature [absolute numbers # and percentage %], age in years (y), age pyramid y: [Age in year], x: [number of patients]. **(B)** Diagnostic methods [absolute numbers#] and results [absolute numbers#]. **(C)** Results in all patients UV, solved, and unsolved [absolute numbers and percentage]. **(D)** Turnaround time, rain cloud plots, y: [time d], x: P, SE, TE.

**Figure 2:**
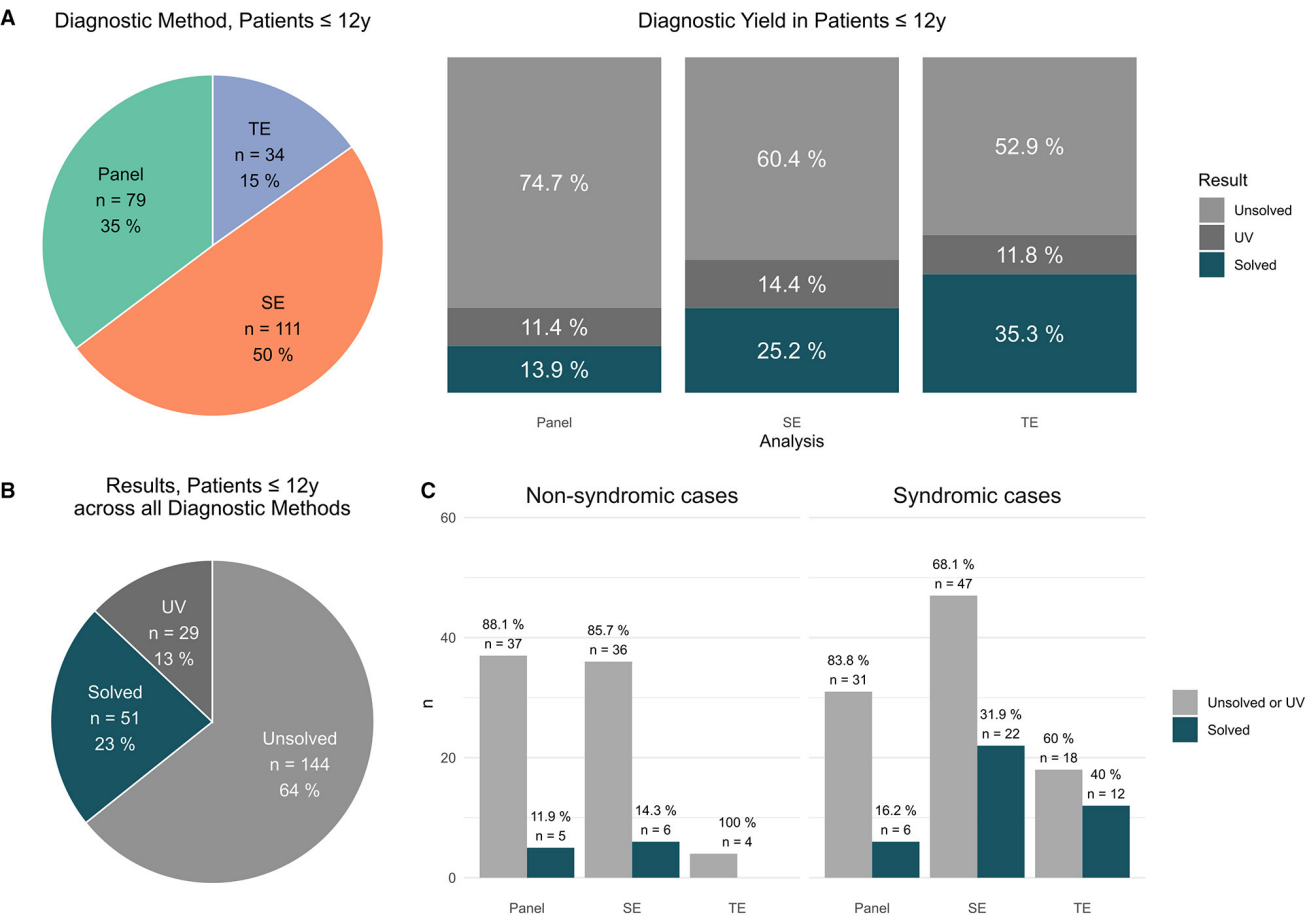
Findings in patients ≤ 12 y. **(A)** Distribution of applied sequencing methods in patients ≤ 12 y [#]; results per applied method in patients ≤ 12 y [unsolved vs. solved vs. UV #]. **(B)** Total results in patients ≤ 12y [unsolved vs. solved vs. UV #]. **(C)** Syndromic cases: Performed analysis (panel vs. exom vs. trio) vs. positive results (solved cases) and non-syndromic cases: Performed analysis (panel vs. exom vs. trio) vs. positive results (solved cases).

**Figure 3:**
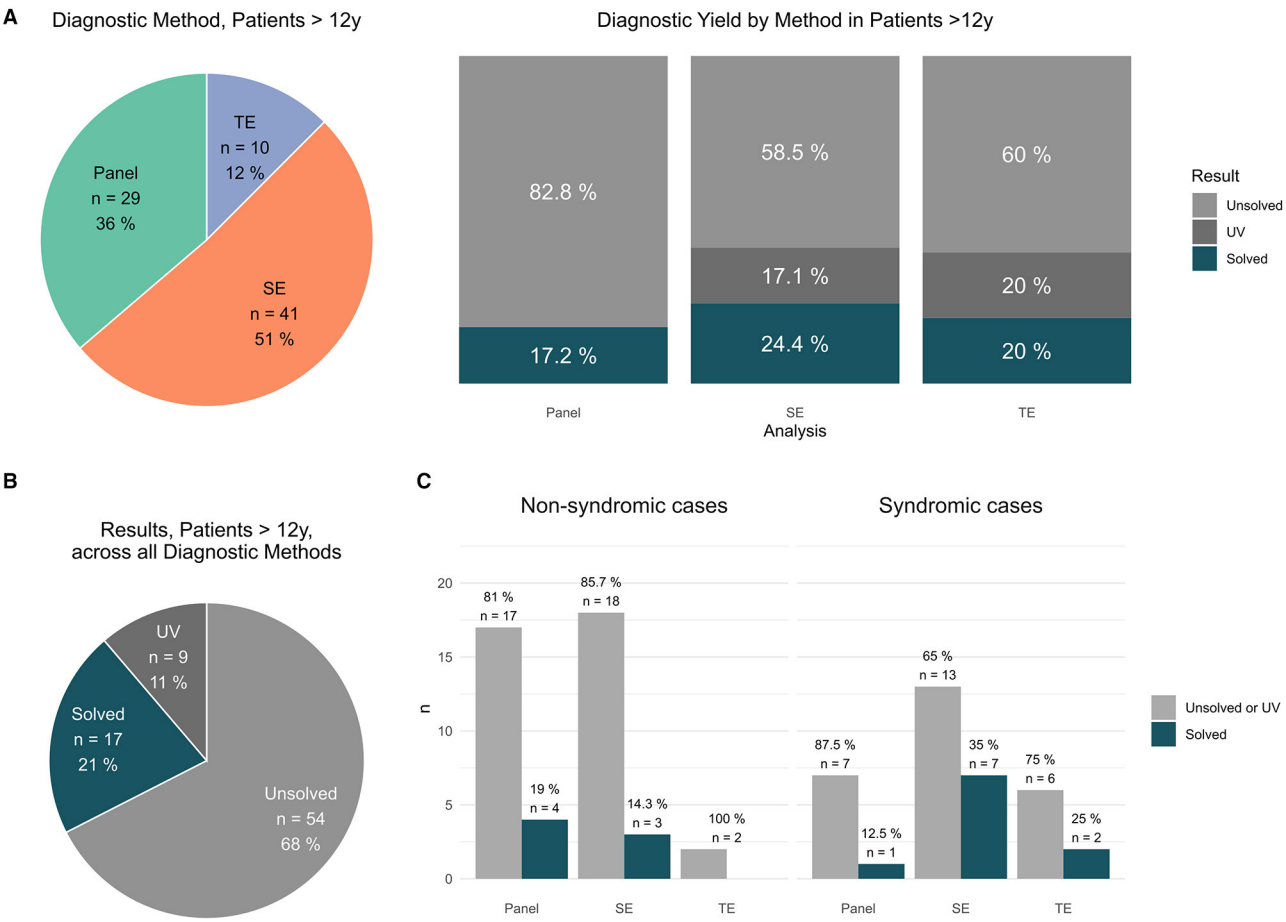
Findings in patients >12 y. **(A)** Distribution of applied sequencing methods in patients > 12 y [#]; results per applied method in patients > 12 y [unsolved vs. solved vs. UV #]. **(B)** Total results in patients >12 y [unsolved vs. solved vs. UV #]. **(C)** Syndromic cases: Performed analysis (panel vs. exom vs. trio) vs. positive results (solved cases) and non-syndromic cases: Performed analysis (panel vs. exom vs. trio) vs. positive results (solved cases).

A total of 304 patients (or 317 returned results, i.e., several VUS per patient possible) were evaluated (Figure 1A). The age pyramid (Figure 1A) showed a progressive decline in patient numbers per year until ~20 years of age. An arbitrary separation was chosen to separately evaluate patients ≤ 12 y (infants to young children) vs. patients > 12 y (adolescents—adults) (Figures 2, 3). The cohort patients ≤ 12 y comprised 224 patients, (74% of all patients, female 44%; syndromic cases 61%) (Figure 2). The cohort “patients > 12 y” comprised 80 patients (female 46%; syndromic cases 45%) (Figure 3).

Overall, SE was applied in half of all patients (152 cases, 50 %), followed by P (108 cases, 36%) and TE (44 cases, 14%), (Figure 1B). Overall, a pathogenic variant (PV; ACMG classes 4/5) was identified in 22% cases; furthermore, a significant number of patients (12%) carried a clinically suggestive variant of unknown significance (VUS) that was reported (Figure 1C).

In our study, a median turnaround time of 20d was measured (*n* = 296, 8 outlier cropped, median 20d, IQR 21.25, mean 27.06d, SD 18.24). For P sequencing (105 cases, median 30d, IQR 20), SE sequencing (147 cases, median 15d, IQR 15.0) and TE sequencing (44 cases, median 16d, IQR 26.0) were calculated. The Kruskal–Wallis test demonstrated that *P*-value was significantly slower (P vs. SE *p* = 4.9 x 10^−10^ and P vs. TE *p* = 0.026), whereas no significant difference was observed between SE vs. TE [*p* = 0.876 (n.s.)] [cf. (Figure 1D)].

In the cohort patients ≤ 12 y, P (79, i.e., 35%), SE (111, i.e., 50%), and TE (34, i.e., 15%) sequencing were performed (Figure 2A). In detail, PV were identified by P in 11/79 (14% cases) by SE in 28/111 (25% cases) and TE 12/34 (35% cases), respectively (Figure 2A). In total, a PV was identified in 51 patients (23%); furthermore, 37 patients (29 (13%) carried a clinically suggestive VUS that was reported (Figure 2B).

In the cohort patients >12 y, P was performed in 29 (i.e., 36%), SE in 41 (i.e., 51%) and TE in 10 cases (i.e., 12%) (Figure 3A). In detail, PV were identified by P in 5/29 (17% cases), by SE in 10/41; (24% cases) and by TE in 2/10 (20% cases), respectively (Figure 3A). In total, a PV was identified in 17 patients (21%); furthermore, 9 patients (11%) carried a clinically suggestive VUS that was reported (Figure 3B).

The average diagnostic yield in patients ≤ 12 y was higher compared to patients >12y cf. Figure 2B vs. Figure 3B.

The highest yields for a definite diagnosis (PV) were achieved within in patients ≤ 12 y by TE [PV in 12 of 34 TE (35%)] (Figure 2A) and as part of this group in patients with a syndromic phenotype [PV in 12 of 30 TE (40%)] (Figure 2C).

**Figure 4:**
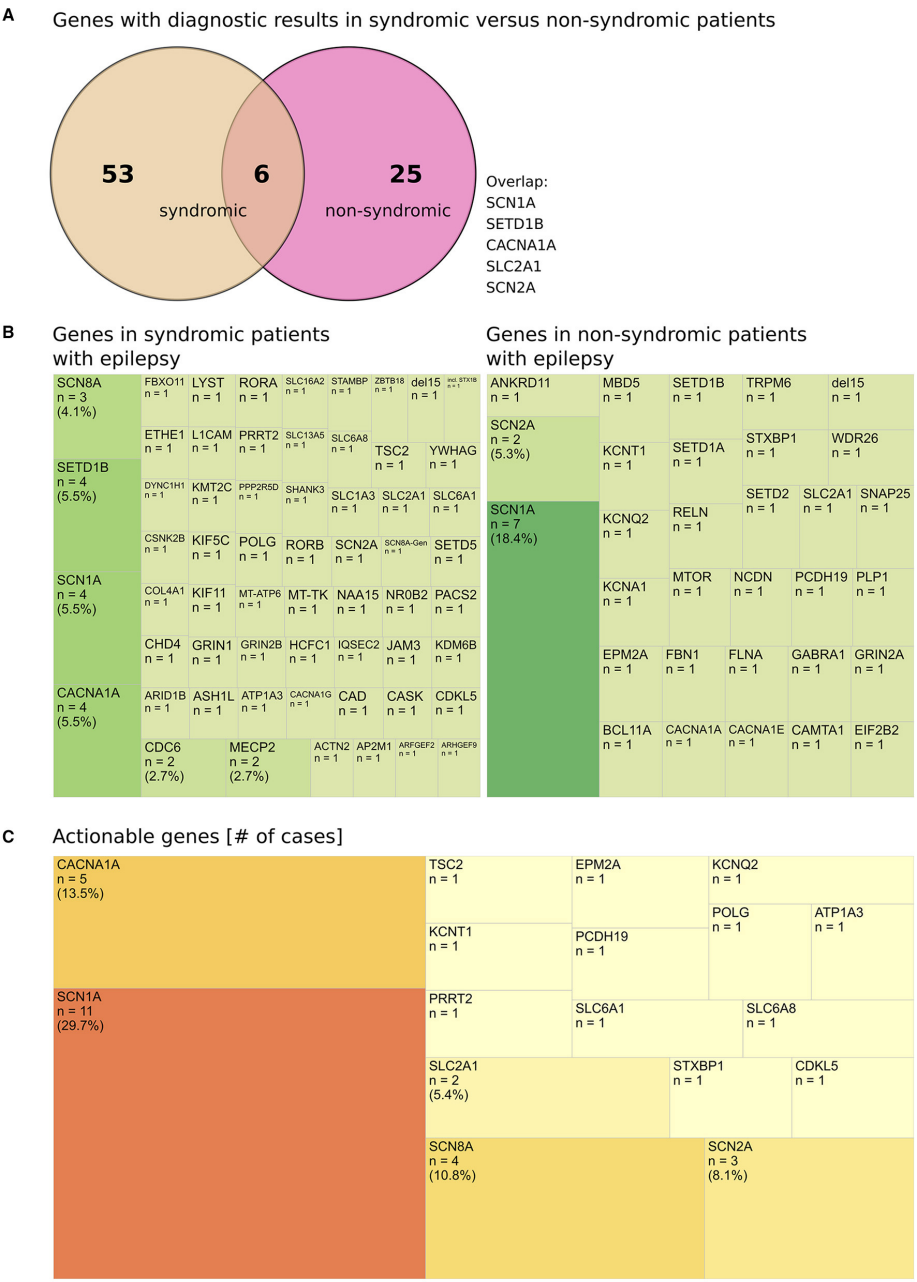
Genes with diagnostic results in syndromic vs. non-syndromic patients. **(A)** Genes with diagnostic results in syndromic vs. non-syndromic patients, (diagnostic: solved or meaningful UV that was reported). **(B)** Heterogeneity (demonstrated by treemap according to material and methods) of genes in syndromic patients with epilepsy and non-syndromic patients with epilepsy. **(C)** Actionable genes (# cases per gene) with diagnostic findings identified in all patients.

Diagnostic findings in syndromic vs. non-syndromic patients revealed a significant overlap of frequent causes of monogenic epilepsies including *SCN1A, CACNA1A*, and *SETD1B* (Figure 4A), thereby confirming the heterogeneity of the associated conditions and demonstrating the variety of causative genes in syndromic epilepsy (Figure 4B).

The top mutated genes (≥3 PV/VUS per gene) were implied in 10.7% of all cases (28/317 returned results) or in 25.7% (28/109) of diagnostic results (cases with a PV or a meaningful VUS which was reported). The majority of genes (75/109 of diagnostic results) was uniquely affected in a single patient (69% % of diagnostic results) (Supplementary Table 2).

For 52/317 reported results, basic clinical information comprising the statement “epilepsy” or “seizure” only was provided. In the majority of 265 of 317 cases, extended clinical information was available. This included but was not limited to semiology of seizures [multi responses possible: neonatale seizure (6 cases), generalized seizures (82 cases), focal seizures (50 cases), absence (32 cases), febril seizures (28 cases)], epilepsy refractors to therapy (7 cases), EEG pathologies (31 cases), cMRI pathologies (37cases, including 8 cases of cortical dysplasia), or additional features [dysmorphology (75 cases), and neurodevelopmental delay (166 cases)] (Table Frontiers_patient data). Patients with extended vs. basic clinical information had a greater tendency to be solved (25 vs. 14%) and a lower tendency for UV (10 vs. 29%), data not shown. Individual follow-up information is not available for this cohort.

Finally, a high proportion of clinically actionable genes was identified among our patient cohort [PV/VUS) (12) plus POLG gene] (Supplementary Table 3). Of 83 genes identified to carry a pathogenic variant or a meaningful finding, 17 genes (18.3%) are considered actionable (Supplementary Table 3; Figure 4C). This accounts for *n* = 37 patient cases in our study and equals to 11.7% (37/317) of all reported results or 33.9% (37/109) of reported PV/VUS results. In detail, in patients ≤ 12 y, actionable genes were implied in 11.1% (26/235) of all reported results or 29.5% (26/88) of PV/VUS reported results. In patients >12 y, actionable genes were implied in 13.4% (11/82) of all reported results or 40.7% (11/27) of PV/VUS reported results (Supplementary Table 3).

## 4 Discussion

Epilepsy can be a symptom of an underlying syndrome or the only clinical symptom. In Germany, 0.6% of the population is affected by epilepsy (13), amounting to ~ 500,000 patients in Germany. The total age-adjusted annual incidence rate in children 1–15 years was estimated to be 60/100,000 (95% confidence interval, 42–84), with the highest incidence in the first year of life (146/100,000). No significant difference in incidence between boys and girls was found (14).

The paradigm of idiopathic epilepsy has been greatly challenged since the 1980s. Previously, epilepsy was either termed idiopathic in ~ 75% of cases, meaning a causative (i.e., anatomical) lesion could not be identified or symptomatic (e.g., trauma, stroke, neoplasm, infection, congenital lesion, and asphyxia associated) (15).

Today, 70–75% of epilepsies are assumed to be caused by monogenic PV (familial, *de novo*), complex inheritance traits, or modifier and susceptibility alleles, of which the latter three traits are not yet amenable to routine diagnostics. The identification of associated genes has been greatly facilitated by next-generation sequencing technologies, leading to a swift increase of novel candidate genes from 2011 onwards. Most of the candidate genes fall into two categories: coding for ion channels or synaptic proteins. Epilepsy is a network pathology with a disturbed balance between excitation and inhibition.

We set out to prospectively identify the proportion of genetic etiologies of epilepsy in a large-scale, real-world scenario of diagnostic laboratories in Germany (molecular results, phenotype (syndromic and non-syndromic), and sequencing methods).

### 4.1 Overall diagnostic yield

In our study PV, (ACMG classes 4 out of 5) were identified in 22% cases and VUS (ACMG class 3) was identified in 12% cases (Figure 1B). The average diagnostic yield in patients ≤ 12 y was higher compared to patients >12y cf. Figure 2B vs. Figure 3B. This effect was not as pronounced as previously assumed possibly due to small numbers of adult patients and clustering of patients into age groups below and above 12 y, thereby assigning adolescents to the older age group. Interestingly, no PV/VUS unique for patients > 12 y were identified, that did not also occur in the younger age group ≤ 12 y (Supplementary Table 4), suggesting that monogenic epilepsies are diseases that manifest from childhood onwards. A specific adult genotype was not apparent in this study.

This is in accordance with testing yields between 15 and 47% in children in the US (16).

### 4.2 Higher diagnostic yield and less VUS by TE in younger patients

The highest diagnostic yields were achieved within the age group ≤ 12 y by TE [12 of 34 TE (35%) with a PV, Figure 2A] and as part of this group in patients with a syndromic phenotype [12 of 30 TE (40%) with a PV, Figure 2C], suggesting that young age and syndromic phenotype are associated with a high likelihood of a causative monogenic disorder. An overlapping finding is that patients with a comprehensively reported phenotype (extended vs. basic clinical information) had a greater tendency to be solved (25 vs. 14%) and a lower tendency for UV (10 vs. 29%) although this includes patients with a syndromic phenotype.

In addition, TE was associated with less VUS (4 out of 34; 12% of cases) compared to SE (VUS 16/c111; 14 %), within this age group ≤ 12 y, showing that TE is more likely to deliver conclusive results (and less clinical uncertainty).

### 4.3 Turnaround time of different NGS methods

Critically ill patients, e.g., patients with neonatal and infantile onset epilepsies benefit from early, molecular diagnosis to inform treatment decisions and to improve prognosis. As defined by D'Gama et al. (17), rapid sequencing can deliver a molecular diagnosis within weeks and ultra-rapid sequencing within days. In keeping with this, routine diagnostic testing in this cohort is able to provide rapid sequencing solutions within <4 weeks; if urgent testing was requested, ultra-rapid results could be provided (see Figure 1D). Additionally, a genetic reevaluation of existing data can also provide molecular findings within days (ultra-rapid).

Clinical urgency can and does considerably speed-up molecular diagnostics. SE (15d) and TE (16d) are considerably quicker than P (20d), and SE and TE are both ordered in more severe cases, e.g., critically ill patients, DEE, neonatal seizures, and syndromic patients. As it is the case in cancer genetics, if therapeutically relevant, a written report can be routinely provided within 10 workdays even with today's routine short read NGS protocols [cf. (Figure 1D)]. The outliers (>100 d, Figure 1D) were caused by the fact that in these patients the respective test (for which the result was reported during this cross-sectional study) was ordered many days after the sample entry; this is why these outliers were excluded from the statistical evaluation to avoid skewed results.

### 4.4 High proportion of actionable findings still underestimates the clinical utility

In 17 out of 26 actionable genes, diagnostic variants (PV or VUS) were identified in our cohort, providing information regarding (1) antiseizure medication (ASM) indication, (2) ASM contraindication, (3) metabolic treatment, or (4) surgical treatments. For example, the diagnosis of a SCN1A-associated seizure disorders can potentially trigger all four modes of action (1–4). In some cases, information regarding the treatment of other allelic disorders will be available (e.g., acetazolamide as treatment for migraine or ataxia in CACNA1A-associated conditions or flunarizine as treatment for ATP1A3- associated alternating hemiplegia of childhood, both conditions can feature seizures). This perspective of actionability centers on therapeutical decisions only. It might therefore underestimate the true utility of diagnostic findings (in our study and in general) since other aspects of diagnostic findings are neglected, e.g., impact on the clinical workup (e.g., avoidance of further invasive testing) or the impact on counseling on patients and families.

### 4.5 High proportion of actionable findings in older patients

To date, older patients with epilepsy are less likely to receive diagnostic testing and especially less likely to receive TE [cf. (Figure 2A) (TE: 34; 15% of patients ≤ 12 y) vs. Figure 3A (TE: 10; 12% patients > 12 y of all studies)], hinting at a general problem of transition into adult medicine associated with reduced medical attention, supportive care, and loss of parental surveillance (and parental availability for TE). It might also be due to the misperception that monogenic traits exclusively affect the clinical trajectory of children and that beyond the age of childhood, the impact of monogenic traits is entirely surpassed by polygenic traits and environmental effects.

Interestingly, genetic diagnostics in older patients proved especially successful with respect to actionable findings. In general, a high proportion of genetic findings may offer the possibility to change the management of our patients (at least 33.9% of all returned PV/VUS results). Strikingly, the proportion of actionable findings in patients >12 y [42.3% (11/26) of diagnostic findings (PV/VUS)] surpassed the proportion of actionable findings in patients ≤ 12 y [29.5% (26/88) of diagnostic findings (PV/VUS)], strongly supporting genetic testing in adolescence and adults with epilepsy (Supplementary Tables 3, 4).

Therefore, the Ontario epilepsy implementation task force and others recommend using the transition to adult medicine as an ideal time point to reevaluate “the diagnosis and repeat […] particularly genetic testing, which now can uncover more etiologies than when patients were initially evaluated many years ago” (18). In keeping with that, genetic associations worldwide, including the German Society of Human Genetics (GfH-Stellungnahme zum Rekontaktieren von Patienten 08/2019), advocate periodical reevaluations in patients without a diagnostic finding based on current genetic technology.

### 4.6 Missed opportunities: less diagnostic findings by P sequencing only

The diagnostic advantage of TE over SE over P is most apparent in the age group ≤ 12 y. A PV was indentified by P (11/79; 14% cases), SE (28/111 25 % cases), and TE (12/34; 35 % cases), respectively. In comparison to TE, diagnostic findings hypothetically remained undisclosed in approximately 20% of cases (P) or 9% of cases (SE), respectively.

However, it is an ongoing discussion whether to sequence a confined multigene panel of clearly actionable genes only [cf. (19)] or to choose a broader approach of SE, TE, or genome sequencing (in the near future). *In silico* panel sequencing is based on a virtual set of genes analyzed by SE and TE (or genome). Targeted enrichment of epilepsy genes comprises simultaneous sequencing of genes included in the panel design and is often outdated quickly after its implementation due to the rapid pace of ongoing gene discovery (20). The German AWMF Guideline 022/007 Klasse S1 suggested confined diagnostic testing in epilepsies of unknown etiology by “karyogram, SNP-array, and panel sequencing.” Supportive to this targeted approach is the fact that PV/VUS cluster in a set of genes. From a clinician's perspective, the wish to preclude unwanted incidental findings and to receive a finite list of genes (presumably ruled out) might be understandable.

Although variants in only a small number of genes (e.g., 30) can provide up to 80% of molecular diagnoses (2), diagnostic results comprise PV/UV in frequently as well as in less commonly affected genes (1). In our study, the top candidate genes (≥3 PV/VUS per gene). Supplementary Table 2 provided 25.7% of diagnostic results. This underlines the fact that less commonly affected genes are implied in the remaining majority of 69% (75/109) of uniquely affected genes in this study.

Interestingly, variants of unknown significance that fit the syndromic phenotype were identified in *CDC6* in a patient with syndromic epilepsy by TE. *CDC6* is associated with Meier–Gorlin syndrome, a syndrome with microcephaly, short stature, and neurodevelopmental delay (OMIM# 2246909); however, an association with epileptic encephalopathy has only recently been suggested (21) illustrating the flexibility of TE over P sequencing.

The Genetics Commission of the International League Against Epilepsy (ILAE) recommends considering epilepsy panels only if SE/TE/Genome is not available, or if deeper sequencing of certain genes is indicated, e.g., if mosaicism is suspected (20). This approach optimizes the diagnostic yield and adds value to results for comparable technical sequencing costs. This study highlights the role of the clinical geneticist in the field to counsel healthcare providers as well as patients:

First, although few genes are responsible for numerous diagnoses, still rare variants can bear information of clinical impact. Second, a finite multigene panel is not a bona fide guarantee. Diagnostic uncertainty prevails until a molecular diagnosis has been established. Even then, genetic modifiers might be identified in future with up-to-date databases and novel technologies. Third, the wish “not to know” should not preclude the molecular diagnosis of rare syndromic maladies associated with possibly actionable genes, especially in case these data already exist.

Epilepsy is a disabling ailment. Therefore, we strongly advocate exploiting the full potential of next-generation sequencing to find the best possible answer for patients, families, and healthcare providers.

## Data availability statement

The original contributions presented in the study are included in the article/Supplementary material, further inquiries can be directed to the corresponding authors.

## Ethics statement

The studies involving humans were approved by internal Ethics Committee of the respective diagnostic centers in Germany. The studies were conducted in accordance with the local legislation and institutional requirements. Written informed consent for participation in this study was provided by the participants' legal guardians/next of kin.

## Author contributions

MW: Conceptualization, Investigation, Visualization, Writing—original draft, Writing—review & editing, Data curation, Formal analysis, Project administration. CG: Formal analysis, Visualization, Writing—review & editing. SW: Investigation, Resources, Writing—review & editing, Data curation. SKl: Investigation, Resources, Writing—review & editing, Data curation. BE: Investigation, Resources, Writing—review & editing. PL: Investigation, Resources, Data curation, Writing—review & editing. LH: Investigation, Resources, Writing—review & editing, Data curation. MK: Investigation, Resources, Writing—review & editing, Data curation. ML: Investigation, Resources, Writing—review & editing. MD: Writing—review & editing. JJ: Data curation, Investigation, Resources, Writing—review & editing. BS: Data curation, Investigation, Resources, Writing—review & editing. KH: Data curation, Investigation, Resources, Writing—review & editing. RG: Data curation, Investigation, Resources, Writing—review & editing. SKn: Data curation, Investigation, Resources, Writing—review & editing. AT: Investigation, Resources, Writing—review & editing. AA: Resources, Writing—review & editing. TN: Conceptualization, Investigation, Methodology, Project administration, Resources, Supervision, Writing—original draft, Writing—review & editing.

## Supplementary material

Supplementary Table 1 Actionable genes in epilepsy. List of genes as published by McKnight et al. (6).

Supplementary Table 2 Top mutated genes in epilepsy, actionability, and associated number of cases. Contains a list of top mutated genes in epilepsy and the associated (cumulative) number of cases in this study (complete study), contains a list of actionable genes identified in all patients, patients ≤ 12 y and >12 y.

Supplementary Table 3 Actionability and associated number of cases. Detailed analysis of genes identified in all patients, patients ≤ 12 y and >12 y.

Supplementary Table 4 Overlap of genes identified in patients ≤ 12 y and >12 y. Contains of list of genes that were identified only in patients ≤ 12 y, (70 genes) both in patients ≤ 12 y and >12 y (13 genes) and only in patients >12 y (none).

Supplementary Table 5 Patient data. Contains a summary of patient data. Case-wise description including analysis, results, genes, clinical information, seizure semiology, EEG pathology, therapy response, NDD, dysmorphology, and cMRI findings.
