# Supplementary material for: Prospective evaluation of NGS-based sequencing in epilepsy patients: results of seven NASGE-associated diagnostic laboratories
Source: Front Neurol. 2023 Dec 6;14:1276238. doi: 10.3389/fneur.2023.1276238 (PMC10731269; doi:10.3389/fneur.2023.1276238)
Supplement: Supplementary Table 5 — Patient data. Contains a summary of patient data. Case-wise description including analysis, results, genes, clinical information, seizure semiology, EEG pathology, therapy response, NDD, dysmorphology, and cMRI findings. [file Data_Sheet_2.docx]

**Clinical features of patients**|

SE: single exome; TE: trio exome; P: panel testing; CNV: copy number variant; VUS: variant of unknown significance; NDD: neurodevelopmental delay; +: feature present; no: feature excluded

| **Patient** | **Analysis** | **Result** | **Gene** | **Clinical information** | **Seizures** | | | | | | **EEG pathology** | **Therapy response** | **NDD** | **Dysmorphologic features** | **cMRI pathology** |
| --- | --- | --- | --- | --- | --- | --- | --- | --- | --- | --- | --- | --- | --- | --- | --- |
|  |  |  |  |  | all | neonatal | grand mal | focal | absence | febrile |  |  |  |  |  |
| 1 | SE | VUS | SETD2 | basic | + |  |  |  |  |  |  |  |  |  |  |
| 2 | SE | unsolved |  | basic | + |  |  |  |  |  |  |  |  |  |  |
| 3 | SE | unsolved |  | extensive | + |  |  |  |  |  |  |  | + |  |  |
| 4 | SE | unsolved |  | extensive | + |  |  | + |  |  | + |  |  | + |  |
| 5 | SE | unsolved |  | extensive | + |  |  | + |  |  |  |  |  |  |  |
| 6 | SE | unsolved |  | basic | + |  |  |  |  |  |  |  |  |  |  |
| 7 | SE | unsolved |  | extensive | + |  |  | + |  |  |  |  |  | + | + |
| 8 | SE | solved | STXBP1 | extensive | + | + | + | + |  |  |  |  | + |  | + |
| 9 | SE | solved | KIF5C | extensive | + | + |  |  |  |  |  |  |  | + | + |
| 10 | SE | solved | JAM3 | extensive | + | + |  |  |  |  |  | + |  | + | + |
| 11 | SE | VUS | SNAP25 | extensive | + | + |  | + |  |  | + |  | + |  |  |
| 12 | SE | solved | SLC13A5 | extensive | + |  |  |  |  |  |  |  | + |  | + |
| 13 | SE | unsolved |  | extensive | + | + | + |  | + |  |  |  |  |  |  |
| 14 | SE | solved | CSNK2B | extensive | + |  | + | + |  |  |  |  | + |  |  |
| 15 | SE | solved | ANKRD11 | extensive | + |  | + |  |  |  |  |  |  |  |  |
| 16 | SE | unsolved |  | basic | + |  |  |  |  |  |  |  |  | + |  |
| 17 | SE | unsolved |  | extensive | + |  | + |  |  | + |  |  | + | + |  |
| 18 | SE | unsolved |  | extensive | + | + |  |  |  |  |  |  |  |  |  |
| 19 | SE | unsolved | TREX1 | extensive | + |  |  | + |  |  |  |  |  |  | + |
| 20 | SE | unsolved | POLG | extensive | + |  |  |  | + |  |  |  | + |  | + |
| 21 | SE | unsolved | PNKP | extensive | + |  |  |  |  | + |  |  |  |  | + |
| 22 | SE | unsolved |  | extensive | + |  |  |  |  | + |  |  |  |  |  |
| 23 | SE | unsolved |  | extensive | + |  |  |  |  |  |  |  | + | + | + |
| 24 | SE | unsolved |  | extensive | + |  |  |  |  |  |  |  | no |  |  |
| 25 | SE | solved | RORA | extensive | + |  |  |  |  |  |  |  | + |  |  |
| 26 | SE | solved | PRRT2 | extensive | + |  |  |  |  |  |  |  | + |  |  |
| 27 | SE | solved | NAA15 | extensive | + |  |  |  |  |  |  |  | + |  |  |
| 28 | SE | solved | MT-ATP6 | extensive | + |  |  |  |  |  |  |  | + | + |  |
| 29 | SE | solved | MECP2 | extensive |  |  |  |  |  |  | + |  | + |  |  |
| 30 | SE | VUS | MBD5 | extensive | + |  |  |  |  |  |  |  |  |  |  |
| 31 | SE | solved | KCNA1 | extensive | + |  |  |  | + |  |  |  | + |  |  |
| 32 | SE | VUS | CACNA1G | extensive | + |  |  |  |  |  |  |  | + |  |  |
| 33 | SE | unsolved |  | extensive | + |  | + |  |  |  |  |  |  |  |  |
| 34 | SE | VUS | ZBTB18 | extensive | + |  |  |  |  |  | + |  | + | + |  |
| 35 | SE | solved | SETD1B | extensive | + |  |  |  | + |  |  |  |  |  |  |
| 36 | SE | unsolved | CTCF | extensive | + |  |  | + |  |  | + |  | + | + | + |
| 37 | SE | VUS | GRIN2B | extensive | + |  | + | + |  |  |  |  | + | + |  |
| 38 | SE | solved | RORB | extensive | + |  | + |  |  |  |  |  | + |  |  |
| 39 | SE | solved | SETD1B | extensive | + |  | + |  |  |  |  |  | + |  |  |
| 40 | SE | VUS | WDR26 | extensive | + |  | + |  | + |  |  |  | + |  |  |
| 41 | SE | VUS | SETD1A | basic | + |  |  |  |  |  |  |  |  |  |  |
| 42 | SE | unsolved |  | extensive | + |  | + |  |  | + |  |  | + | + |  |
| 43 | SE | unsolved |  | extensive | + |  |  | + |  |  |  |  |  | + | + |
| 44 | SE | unsolved |  | extensive | + |  | + | + |  |  | + |  |  |  |  |
| 45 | SE | unsolved |  | extensive | + |  |  |  | + | + | + |  |  |  |  |
| 46 | SE | solved | TSC2 | extensive | + |  | + |  |  |  |  |  | + | + |  |
| 47 | SE | VUS | SCN8A | extensive | + |  | + |  |  |  |  |  | + |  |  |
| 48 | SE | unsolved |  | extensive | + |  | + |  |  |  |  |  |  |  |  |
| 49 | SE | unsolved | POLG | basic | + |  |  |  |  |  |  |  |  | + |  |
| 50 | SE | unsolved | VPS4A | extensive | + |  | + |  |  |  | + |  | + | + | + |
| 51 | SE | solved | CNV | extensive | + |  |  |  |  | + |  |  | + |  |  |
| 52 | SE | unsolved |  | extensive | + |  | + |  |  |  | + |  | + | + |  |
| 53 | SE | unsolved |  | extensive | + |  | + |  |  |  |  |  |  |  |  |
| 54 | SE | unsolved |  | extensive | + |  | + |  |  |  | + |  |  |  |  |
| 55 | SE | unsolved |  | extensive | + |  | + |  |  |  |  |  |  |  |  |
| 56 | SE | unsolved | VPS13B | extensive | + |  |  |  |  |  |  |  | + | + |  |
| 57 | SE | unsolved |  | extensive | + |  | + |  |  |  |  |  | + | + |  |
| 58 | SE | unsolved |  | extensive | + |  |  |  |  |  |  |  | + |  |  |
| 59 | SE | solved | ARID1B | extensive | + |  |  |  |  |  |  |  | + |  |  |
| 60 | SE | unsolved |  | extensive | + |  |  |  |  |  |  |  | + |  |  |
| 61 | SE | unsolved |  | extensive | + |  |  |  | + |  |  |  |  |  |  |
| 62 | SE | unsolved |  | extensive | + |  |  |  |  |  |  |  | + |  |  |
| 63 | SE | unsolved |  | extensive | + |  |  | + |  |  |  |  | + |  |  |
| 64 | SE | unsolved |  | extensive | + |  |  |  | + |  |  |  |  |  |  |
| 65 | SE | unsolved |  | extensive | + |  | + |  |  |  |  |  | + |  |  |
| 66 | SE | VUS | SETD1B | extensive | + |  |  |  |  |  |  |  | + | + |  |
| 67 | SE | unsolved | KCNQ2 | extensive | + |  | + |  |  |  |  |  |  |  |  |
| 68 | SE | solved | CACNA1A | basic | + |  |  |  |  |  |  |  |  |  |  |
| 69 | SE | solved | ARHGEF9 | extensive | + |  | + | + |  |  |  |  | + | + |  |
| 70 | SE | unsolved |  | extensive | + |  |  |  | + |  | + |  |  |  |  |
| 71 | SE | unsolved |  | extensive | + |  |  |  |  |  | + |  | + |  |  |
| 72 | SE | unsolved |  | extensive | + |  |  | + |  |  |  |  |  |  |  |
| 73 | SE | unsolved |  | extensive | + |  |  |  |  |  |  |  | + |  |  |
| 74 | SE | solved | SETD5 | extensive | + |  |  |  |  |  |  |  | + | + |  |
| 75 | SE | unsolved | RAB3GAP1 | extensive | + |  |  |  |  |  |  |  | + | + |  |
| 76 | SE | unsolved | GNB1 | extensive | + |  | + |  |  |  |  |  |  |  |  |
| 77 | SE | solved | BCL11A | extensive | + |  |  | + |  |  |  |  | + |  |  |
| 78 | SE | unsolved |  | extensive | + |  |  | + |  |  |  |  | + | + |  |
| 79 | SE | unsolved |  | extensive | + |  | + | + |  | + |  |  | + | + |  |
| 80 | SE | VUS | SETD1B | extensive | + |  | + |  |  |  |  |  | + |  |  |
| 81 | SE | VUS | SETD1B | extensive | + |  | + |  |  |  |  |  | + |  |  |
| 82 | SE | VUS | SCN1A | extensive | + |  |  |  | + | + |  |  |  |  |  |
| 83 | SE | unsolved |  | extensive | + |  | + | + |  |  | + |  |  |  |  |
| 84 | SE | solved | CNV | extensive | + |  | + |  | + |  |  | no |  |  | + |
| 85 | SE | unsolved |  | extensive | + |  |  |  |  | + |  |  |  |  |  |
| 86 | SE | unsolved |  | extensive | + |  |  | + | + |  | + |  |  |  |  |
| 87 | SE | unsolved |  | extensive | + |  |  | + |  |  | + |  | + |  |  |
| 88 | SE | unsolved |  | extensive | + |  |  |  |  |  |  |  | + | + | + |
| 89 | SE | unsolved |  | extensive | + |  |  |  |  |  |  |  | + | + |  |
| 90 | SE | unsolved |  | extensive | + |  | + |  |  |  |  |  | + |  |  |
| 91 | SE | unsolved |  | extensive | + |  | + |  | + |  |  | no |  |  |  |
| 92 | SE | unsolved |  | extensive | + |  |  |  |  |  |  |  | + |  |  |
| 93 | SE | unsolved |  | extensive | + |  | + |  |  |  |  |  | + |  |  |
| 94 | SE | unsolved |  | extensive | + |  |  | + |  |  |  |  |  |  |  |
| 95 | SE | solved | SCN1A | extensive | + |  |  |  |  |  |  |  | + |  |  |
| 96 | SE | unsolved |  | extensive | + |  |  | + |  |  |  |  |  |  |  |
| 97 | SE | unsolved |  | extensive | + |  |  |  |  |  |  |  | + |  | + |
| 98 | SE | unsolved |  | extensive | + |  | + |  | + |  | + |  | + |  |  |
| 99 | SE | unsolved | WASF1 | extensive | + |  | + |  |  |  | + |  | + |  |  |
| 100 | SE | VUS | STAMBP | extensive | + |  |  |  |  |  |  |  | + | + |  |
| 101 | SE | VUS | PLP1 | basic | + |  |  |  |  |  |  |  |  |  |  |
| 102 | SE | VUS | NR0B2 | extensive | + |  |  |  |  |  |  |  | + | + |  |
| 103 | SE | VUS | NCDN | basic | + |  |  |  |  |  |  |  |  |  |  |
| 104 | SE | VUS | LYST | extensive | + |  |  |  |  |  |  |  | + | + |  |
| 105 | SE | solved | KDM6B | extensive | + |  |  |  |  |  |  |  | + | + | + |
| 106 | SE | VUS | CHD4 | extensive | + |  |  |  |  |  |  |  | + | + |  |
| 107 | SE | VUS | CACNA1E | basic | + |  |  |  |  |  |  |  |  |  |  |
| 108 | SE | unsolved |  | extensive | + |  |  |  | + |  |  |  | + | + |  |
| 109 | SE | unsolved |  | basic | + |  |  |  |  |  |  |  |  |  |  |
| 110 | SE | solved | YWHAG | extensive | + |  |  |  |  |  |  |  | + | + |  |
| 111 | SE | solved | SLC6A8 | extensive | + |  | + |  |  |  |  |  | + |  |  |
| 112 | SE | solved | SLC16A2 | extensive | + |  | + |  |  |  |  |  | + | + | + |
| 113 | SE | VUS | DYNC1H1 | extensive | + |  |  |  |  |  |  |  |  | + |  |
| 114 | SE | unsolved | CYFIP2 | extensive | + |  | + |  |  |  |  |  | + |  | + |
| 115 | SE | VUS | ACTN2 | extensive | + |  |  |  |  |  |  |  |  | + |  |
| 116 | SE | unsolved |  | extensive | + |  | + |  |  |  |  |  |  |  |  |
| 117 | SE | unsolved |  | extensive | + |  |  |  |  |  |  |  | + | + |  |
| 118 | SE | VUS | SCN2A | extensive | + |  |  |  |  |  |  |  | + | + | + |
| 119 | SE | unsolved |  | extensive | + |  |  | + |  |  |  |  |  |  | no |
| 120 | SE | unsolved |  | extensive | + |  | + |  |  |  |  |  |  |  |  |
| 121 | SE | unsolved |  | basic | + |  |  |  |  |  |  |  |  |  |  |
| 122 | SE | unsolved | NARS1 | extensive | + |  |  |  | + |  |  |  | + | + |  |
| 123 | SE | VUS | EPM2A | extensive | + |  | + |  |  |  |  |  |  |  |  |
| 124 | SE | solved | CNV | extensive | + |  |  |  | + |  | + |  |  |  |  |
| 125 | SE | unsolved |  | extensive | + |  |  | + | + |  |  |  |  |  |  |
| 126 | SE | unsolved |  | extensive | + |  |  |  |  |  |  |  | + |  | + |
| 127 | SE | VUS | POLG | basic | + |  |  |  |  |  |  |  |  | + |  |
| 128 | SE | solved | ATP1A3 | extensive | + |  |  |  |  | + |  |  | + | + |  |
| 129 | SE | VUS | AP2M1 | basic | + |  |  |  |  |  |  |  |  | + |  |
| 130 | SE | unsolved |  | extensive | + |  |  | + |  |  |  |  |  |  | + |
| 131 | SE | unsolved |  | extensive | + |  | + |  | + |  |  |  | + |  |  |
| 132 | SE | solved | SCN8A | extensive | + |  | + |  |  |  |  |  | + | + |  |
| 133 | SE | VUS | MTOR | extensive | + |  |  | + |  |  |  |  | + |  | + |
| 134 | SE | solved | FBXO11 | extensive | + |  |  |  |  |  |  | no | + |  |  |
| 135 | SE | unsolved |  | extensive | + |  |  | + |  |  |  |  |  |  |  |
| 136 | SE | unsolved |  | extensive | + |  | + | + |  |  |  |  | + |  | + |
| 137 | SE | unsolved |  | extensive | + |  | + |  |  |  |  |  |  |  |  |
| 138 | SE | unsolved |  | basic | + |  |  | + |  |  |  |  |  |  | + |
| 139 | SE | VUS | KMT2C | extensive | + |  |  |  |  |  |  |  | + |  | + |
| 140 | SE | unsolved |  | extensive | + |  |  |  |  |  | + |  |  |  |  |
| 141 | SE | solved | PPP2R5D | extensive | + |  | + |  |  |  |  |  | + | + |  |
| 142 | SE | solved | FBN1 | basic | + |  |  |  |  |  |  |  |  |  |  |
| 143 | SE | unsolved |  | basic | + |  |  |  |  |  |  |  |  |  |  |
| 144 | SE | unsolved |  | extensive | + |  |  |  |  |  |  |  | + | + |  |
| 145 | SE | unsolved |  | extensive | + |  | + | + |  |  |  | no | + |  |  |
| 146 | SE | solved | SHANK3 | extensive | + |  | + |  |  |  |  |  | + |  |  |
| 147 | SE | unsolved |  | extensive | + |  |  | + |  |  |  |  |  |  |  |
| 148 | SE | VUS | SLC2A1 | extensive | + |  | + |  |  |  |  |  | + |  |  |
| 149 | SE | unsolved |  | extensive | + |  |  | + |  |  |  | no | no |  | no |
| 150 | SE | unsolved |  | extensive | + |  | + |  |  |  |  |  |  |  |  |
| 151 | SE | solved | CNV | extensive | + |  | + |  |  |  |  |  | + |  |  |
| 152 | SE | VUS | CNV | extensive | + |  |  |  |  |  |  |  | + |  |  |
| 153 | SE | unsolved |  | extensive |  |  | + |  |  |  | + |  | + |  |  |
| 154 | SE | unsolved | BTD | extensive | + |  |  |  |  |  |  |  | + |  | no |
| 155 | SE | unsolved |  | extensive | + |  |  |  |  |  |  |  |  |  |  |
| 156 | SE | solved | SCN1A | extensive | + |  | + |  |  |  |  |  | + |  |  |
| 157 | SE | solved | CNV | extensive | + |  |  | + |  |  |  | no | + |  | no |
| 158 | SE | unsolved |  | extensive | + |  |  | + |  |  |  |  |  |  |  |
| 159 | SE | unsolved |  | extensive | + |  | + | + |  |  |  |  |  |  |  |
| 160 | p | VUS | SCN2A | basic | + |  |  |  |  |  |  |  |  |  |  |
| 161 | P | solved | SCN2A | basic | + |  |  |  |  |  |  |  |  |  |  |
| 162 | P | unsolved | GRIN2D | extensive | + |  | + |  |  |  |  |  |  |  |  |
| 163 | p | unsolved |  | basic | + |  |  |  |  |  |  |  |  |  |  |
| 164 | P | unsolved |  | extensive | + |  | + |  |  |  |  |  |  |  |  |
| 165 | P | unsolved |  | basic | + |  |  |  |  |  |  |  |  |  |  |
| 166 | P | unsolved |  | extensive | + |  | + |  |  |  |  |  |  |  |  |
| 167 | P | unsolved |  | basic | + |  |  |  |  |  |  |  |  |  |  |
| 168 | P | unsolved |  | extensive | + |  |  | + |  |  | + |  |  | + |  |
| 169 | P | unsolved |  | extensive | + |  |  |  |  |  |  |  |  |  | + |
| 170 | P | solved | CASK | basic | + |  |  |  |  |  |  |  |  |  |  |
| 171 | P | solved | CDKL5 | extensive | + |  | + |  |  |  |  |  |  |  |  |
| 172 | P | unsolved |  | extensive | + |  |  |  |  |  |  |  | + |  |  |
| 173 | P | unsolved |  | extensive | + |  |  |  |  |  |  |  | + | + |  |
| 174 | p | VUS | SCN1A | basic | + |  |  |  |  |  |  |  |  |  |  |
| 175 | P | solved | SCN1A | extensive | + |  |  |  |  | + |  |  | + |  |  |
| 176 | P | unsolved |  | extensive | + |  |  |  |  | + |  |  |  |  |  |
| 177 | P | unsolved |  | extensive | + |  |  | + |  |  |  |  |  |  |  |
| 178 | P | unsolved |  | extensive | + |  |  |  |  | + |  |  |  |  |  |
| 179 | P | unsolved |  | extensive | + |  | + |  |  | + |  |  |  |  |  |
| 180 | P | unsolved |  | extensive | + |  |  |  |  | + |  |  |  |  |  |
| 181 | P | unsolved |  | basic | + |  |  |  |  |  |  |  |  |  |  |
| 182 | P | unsolved |  | extensive | + |  |  |  |  | + |  |  |  |  |  |
| 183 | P | unsolved |  | extensive | + |  |  |  |  | + |  |  |  |  |  |
| 184 | P | solved | SCN1A | extensive | + |  |  |  |  | + |  |  | + |  |  |
| 185 | P | solved | FLNA | extensive | + |  |  |  |  |  |  |  |  |  |  |
| 186 | P | unsolved |  | extensive | + |  |  |  |  |  |  |  | + |  |  |
| 187 | P | VUS | SCN1A | extensive | + |  |  |  |  | + |  |  |  |  |  |
| 188 | p | VUS | KCNT1 | basic | + |  |  |  |  |  |  |  |  |  |  |
| 189 | P | unsolved |  | extensive | + |  |  |  |  |  |  |  | + |  |  |
| 190 | P | unsolved |  | extensive | + |  |  |  |  | + |  |  |  |  |  |
| 191 | P | unsolved |  | basic | + |  |  |  |  |  |  |  |  |  |  |
| 192 | P | unsolved |  | extensive | + |  |  |  |  | + |  |  | + |  |  |
| 193 | P | solved | IQSEC2 | extensive | + |  |  |  |  |  |  |  | + |  |  |
| 194 | P | unsolved |  | basic | + |  |  |  |  |  |  |  |  |  |  |
| 195 | P | unsolved |  | basic | + |  |  |  |  |  |  |  | + |  |  |
| 196 | P | unsolved |  | extensive | + |  |  |  |  |  | + |  | + |  |  |
| 197 | P | unsolved |  | extensive | + |  |  |  | + |  |  |  |  |  |  |
| 198 | P | unsolved |  | extensive | + |  |  |  | + | + |  |  | + |  |  |
| 199 | p | unsolved |  | extensive | + |  |  | + | + |  |  |  | + |  |  |
| 200 | P | VUS | SLC2A1 | extensive | + |  | + |  |  |  |  |  |  |  |  |
| 201 | P | unsolved |  | extensive | + |  |  |  |  |  |  |  | + |  |  |
| 202 | P | unsolved |  | extensive | + |  |  |  |  | + | + |  |  |  |  |
| 203 | P | unsolved |  | extensive | + |  |  |  |  |  |  |  | + |  |  |
| 204 | P | unsolved |  | basic | + |  |  |  |  |  |  |  |  |  |  |
| 205 | P | unsolved |  | extensive | + |  |  |  |  |  |  |  | + |  |  |
| 206 | P | unsolved |  | extensive | + |  |  |  | + |  |  |  | + |  |  |
| 207 | P | unsolved |  | extensive | + |  |  |  | + |  |  |  | + |  |  |
| 208 | P | solved | GRIN2A | extensive | + |  |  | + |  |  |  |  |  |  |  |
| 209 | P | unsolved |  | basic | + |  |  |  |  |  |  |  |  |  |  |
| 210 | P | unsolved |  | extensive | + |  |  |  |  | + |  |  |  |  |  |
| 211 | P | unsolved |  | extensive | + |  |  |  |  |  | + |  | + |  | + |
| 212 | P | unsolved |  | extensive | + |  |  |  | + |  |  |  | + |  |  |
| 213 | P | unsolved |  | extensive | + |  |  |  |  |  |  |  | + |  |  |
| 214 | P | unsolved |  | extensive | + |  |  |  |  |  |  |  | + |  |  |
| 215 | P | unsolved |  | extensive | + |  |  |  |  |  |  |  | + |  |  |
| 216 | P | unsolved |  | extensive | + |  |  |  |  |  |  |  | + | + |  |
| 217 | P | VUS | GABRA1 | basic | + |  |  |  |  |  |  |  |  |  |  |
| 218 | P | unsolved |  | extensive | + |  |  |  |  |  |  |  | + |  |  |
| 219 | P | VUS | SCN8A | basic | + |  |  |  |  |  |  |  |  |  |  |
| 220 | P | unsolved |  | basic | + |  |  |  |  |  |  |  |  |  |  |
| 221 | P | unsolved |  | extensive | + |  |  |  |  |  |  |  | + | + |  |
| 222 | P | unsolved |  | extensive | + |  |  |  |  |  |  |  | + |  |  |
| 223 | P | unsolved |  | extensive | + |  |  |  |  |  |  |  | + |  |  |
| 224 | P | unsolved |  | extensive | + |  |  | + |  |  |  |  |  |  |  |
| 225 | P | unsolved |  | basic | + |  |  |  |  |  |  |  |  |  |  |
| 226 | P | solved | GRIN1 | extensive | + |  |  |  |  |  |  |  | + |  |  |
| 227 | P | unsolved |  | extensive | + |  |  |  | + |  |  |  |  |  |  |
| 228 | P | unsolved |  | extensive | + |  |  |  |  | + |  |  |  |  |  |
| 229 | P | unsolved |  | extensive | + |  |  |  | + |  |  |  | + | + |  |
| 230 | P | solved | SCN1A | extensive | + |  | + |  | + |  | + |  |  |  |  |
| 231 | P | unsolved |  | extensive | + |  |  | + |  |  |  |  |  |  |  |
| 232 | P | unsolved |  | basic | + |  |  |  |  |  |  |  |  |  |  |
| 233 | P | solved | CACNA1A | extensive | + |  |  |  |  |  |  |  | + |  |  |
| 234 | P | unsolved |  | extensive | + |  | + |  | + |  | + |  |  |  |  |
| 235 | P | unsolved |  | extensive | + |  |  |  |  |  |  |  | + |  |  |
| 236 | P | unsolved |  | extensive | + |  |  |  |  |  |  |  | + | + |  |
| 237 | P | unsolved |  | extensive | + |  |  | + |  |  | + |  | + |  |  |
| 238 | p | VUS | RELN | basic | + |  |  |  |  |  |  |  |  |  |  |
| 239 | P | unsolved |  | extensive | + |  |  |  |  |  |  |  | + |  |  |
| 240 | P | VUS | PCDH19 | extensive | + |  | + | + |  |  |  |  |  |  |  |
| 241 | P | unsolved |  | extensive | + |  |  |  |  |  |  |  |  |  |  |
| 242 | P | unsolved |  | extensive | + |  |  | + |  |  |  |  |  |  |  |
| 243 | P | unsolved |  | extensive | + |  |  |  |  |  |  |  | + |  |  |
| 244 | P | unsolved |  | extensive | + |  |  |  |  |  |  |  | + | + |  |
| 245 | P | solved | SCN8A | extensive | + |  |  |  | + |  |  |  | + |  |  |
| 246 | P | unsolved |  | extensive | + |  |  |  |  |  |  |  | + |  |  |
| 247 | P | unsolved |  | extensive | + |  | + |  |  |  |  |  |  |  |  |
| 248 | P | unsolved |  | extensive | + |  |  |  | + |  | + |  |  |  |  |
| 249 | p | unsolved |  | basic | + |  |  |  |  |  |  |  |  |  |  |
| 250 | P | unsolved |  | extensive | + |  |  |  | + |  |  |  |  |  |  |
| 251 | P | unsolved |  | extensive | + |  |  |  |  |  |  |  | + |  |  |
| 252 | P | unsolved |  | extensive | + |  |  |  |  |  |  |  | + |  |  |
| 253 | P | unsolved |  | extensive | + |  |  |  |  |  |  |  | + |  |  |
| 254 | P | solved | SCN1A | extensive | + |  | + |  |  |  |  |  |  |  |  |
| 255 | P | unsolved |  | extensive | + |  |  | + |  |  |  |  |  |  |  |
| 256 | P | unsolved |  | extensive | + |  |  | + |  |  |  |  |  |  |  |
| 257 | P | unsolved |  | basic | + |  |  |  |  |  |  |  |  |  |  |
| 258 | P | solved | TRPM6 | basic | + |  |  |  |  |  |  |  |  |  |  |
| 259 | p | unsolved |  | basic | + |  |  |  |  |  |  |  |  |  |  |
| 260 | P | unsolved |  | basic | + |  |  |  |  |  |  |  |  |  |  |
| 261 | p | solved | SCN1A | basic | + |  |  |  |  |  |  |  |  |  |  |
| 262 | P | solved | KCNQ2 | basic | + |  |  |  |  |  |  |  |  |  |  |
| 263 | P | unsolved |  | basic |  |  |  |  |  |  |  |  |  |  |  |
| 264 | P | unsolved |  | basic | + |  |  |  |  |  |  |  |  |  |  |
| 265 | P | unsolved |  | basic | + |  |  |  |  |  |  |  |  |  |  |
| 266 | P | unsolved |  | basic | + |  |  |  |  |  |  |  |  |  |  |
| 267 | P | unsolved |  | basic | + |  |  |  |  |  |  |  |  |  |  |
| 268 | P | unsolved |  | extensive | + |  | + |  |  |  |  |  |  |  |  |
| 269 | p | unsolved |  | basic | + |  |  |  |  |  |  |  |  |  |  |
| 270 | p | unsolved |  | extensive | + |  | + |  |  |  |  |  |  |  | + |
| 271 | P | unsolved |  | extensive | + |  |  |  |  |  |  |  |  |  |  |
| 272 | TE | solved | ETHE1 | extensive | + |  | + |  |  |  |  |  |  | + |  |
| 273 | TE | unsolved |  | extensive | + |  | + | + |  |  |  |  | + |  |  |
| 274 | TE | solved | CAD | extensive | + |  |  |  |  |  |  |  |  | + | + |
| 275 | TE | unsolved |  | extensive | + |  | + |  |  |  |  |  | + | + | + |
| 276 | TE | solved | PACS2 | extensive | + |  | + |  |  |  |  |  | + | + |  |
| 277 | TE | solved | MT-TK | extensive | + |  |  |  |  |  |  |  | + | + |  |
| 278 | TE | VUS | SCN1A | basic | + |  |  |  |  |  |  |  |  |  |  |
| 279 | TE | VUS | CAMTA1 | basic | + |  |  |  |  |  |  |  |  |  |  |
| 280 | TE | unsolved |  | extensive | + |  |  |  |  |  |  |  | + | + |  |
| 281 | TE | unsolved |  | extensive | + |  | + |  |  |  |  |  |  |  |  |
| 282 | TE | unsolved |  | extensive | + |  | + |  |  |  | + |  | + |  |  |
| 283 | TE | VUS | SLC1A3 | extensive | + |  |  |  |  |  |  |  | + | + |  |
| 284 | TE | solved | CACNA1A | extensive | + |  |  | + |  |  |  |  | + |  |  |
| 285 | TE | solved | SLC6A1 | extensive | + |  | + |  |  |  |  |  | + |  |  |
| 286 | TE | unsolved |  | extensive | + |  |  |  |  |  |  |  | + | + |  |
| 287 | TE | unsolved |  | extensive | + |  | + |  |  |  |  |  | + |  |  |
| 288 | TE | solved | COL4A1 | extensive | + |  |  |  |  |  |  |  | + | + | + |
| 289 | TE | solved | CACNA1A | extensive | + |  |  |  |  |  |  |  | + |  | + |
| 290 | TE | unsolved |  | extensive | + |  |  |  |  |  |  |  | + |  |  |
| 291 | TE | unsolved |  | extensive | + |  |  |  |  |  |  |  | + | + |  |
| 292 | TE | unsolved |  | extensive | + |  | + |  |  |  |  |  | + | + |  |
| 293 | TE | solved | ASH1L | extensive | + |  |  |  |  |  | + |  | + |  |  |
| 294 | TE | solved | ARFGEF2 | extensive | + |  | + |  |  |  |  |  | + | + | + |
| 295 | TE | unsolved |  | extensive | + |  |  |  |  |  |  |  |  |  |  |
| 296 | TE | solved | CACNA1A | extensive | + |  |  |  |  |  |  |  | + |  |  |
| 297 | TE | unsolved |  | extensive | + |  |  |  |  |  |  |  | + |  |  |
| 298 | TE | solved | L1CAM | extensive | + |  |  |  |  |  |  |  |  | + | + |
| 299 | TE | VUS | EIF2B2 | extensive | + |  | + |  |  |  |  |  | + |  |  |
| 300 | TE | unsolved |  | extensive | + |  |  |  |  |  |  |  | + |  |  |
| 301 | TE | unsolved |  | extensive | + |  |  |  |  |  |  |  | + |  |  |
| 302 | TE | VUS | CDC6 | extensive | + |  |  |  |  |  |  |  | + |  |  |
| 303 | TE | VUS | CDC6 | extensive | + |  |  |  |  |  |  |  | + |  |  |
| 304 | TE | unsolved |  | extensive | + |  | + |  |  |  |  |  | + | + | + |
| 305 | TE | unsolved |  | extensive | + |  |  |  |  | + |  |  | + | + |  |
| 306 | TE | unsolved |  | extensive | + |  |  |  |  | + |  |  | + | + |  |
| 307 | TE | unsolved |  | extensive | + |  |  |  |  | + |  |  | + | + |  |
| 308 | TE | unsolved |  | extensive | + |  | + |  |  |  |  |  |  |  |  |
| 309 | TE | unsolved |  | extensive | + |  | + |  |  |  |  |  |  |  |  |
| 310 | TE | solved | MECP2 | extensive | + |  |  |  |  |  |  |  | + |  | no |
| 311 | TE | unsolved |  | extensive | + |  | + | + |  |  |  |  | + | + | no |
| 312 | TE | solved | STX1B | extensive | + |  |  | + |  |  |  |  | + |  |  |
| 313 | TE | unsolved | DARS | extensive | + |  |  |  |  |  | + |  | + | + | + |
| 314 | TE | VUS | HCFC1 | extensive | + |  |  |  |  |  |  |  | + | + |  |
| 315 | TE | VUS | KIF11 | extensive | + |  |  |  |  |  |  |  | + | + | + |
| 316 | TE | unsolved |  | extensive | + |  |  |  |  |  |  | no | + | + |  |
| 317 | TE | unsolved |  | extensive | + |  |  |  |  |  |  |  | + |  |  |
